# Temperature sum models in plant spring phenology studies: two commonly used methods have different fields of application

**DOI:** 10.1093/jxb/erae363

**Published:** 2024-08-27

**Authors:** Rui Zhang, Fucheng Wang, Jinbin Zheng, Lei Chen, Heikki Hänninen, Jiasheng Wu

**Affiliations:** State Key Laboratory of Subtropical Silviculture, Zhejiang A&F University, Hangzhou 311300, China; Key Laboratory of Modern Silvicultural Technology of Zhejiang Province, Hangzhou 311300, China; State Key Laboratory of Subtropical Silviculture, Zhejiang A&F University, Hangzhou 311300, China; Key Laboratory of Modern Silvicultural Technology of Zhejiang Province, Hangzhou 311300, China; State Key Laboratory of Subtropical Silviculture, Zhejiang A&F University, Hangzhou 311300, China; Key Laboratory of Modern Silvicultural Technology of Zhejiang Province, Hangzhou 311300, China; Key Laboratory of Bio-Resource and Eco-Environment of Ministry of Education, College of Life Sciences, Sichuan University, Chengdu, China; State Key Laboratory of Subtropical Silviculture, Zhejiang A&F University, Hangzhou 311300, China; Key Laboratory of Modern Silvicultural Technology of Zhejiang Province, Hangzhou 311300, China; State Key Laboratory of Subtropical Silviculture, Zhejiang A&F University, Hangzhou 311300, China; Key Laboratory of Modern Silvicultural Technology of Zhejiang Province, Hangzhou 311300, China; University of Western Australia, Australia

**Keywords:** Growing degree day, growing degree hour, heat requirement, model realism, process-based phenology models, spring phenology, temperature sum, threshold temperature

**In studies of plant spring phenology, temperature sum models are traditional tools. They are used to quantify plant development in terms of accumulation of temperature-dependent developmental units, such as growing degree hours. A key parameter in these models is the threshold (or base) temperature, representing the lower thermal limit for development to occur. The parameter can be either estimated when the model is fitted to the data or fixed *a priori*. Here we examine the limitations of both methods and identify fields of application for each of them.**

The calculation of various temperature sums has a long history in studies of plant phenology, dating back to the 18th century (Réaumur, 1735; Arnold, 1959; Chuine *et al.*, 2024). Growing degree hour (GDH) or growing degree day models approximate the physiological temperature response of plant development in the spring (Supplementary Fig. S1):


$$\begin{array}{l} Rate\;of\;development\left(T\right)=max\left[\left[\left(\frac{100}{GDH_{crit}}\right)\left(T-T_{thr}\right)\right]\;,0\right] \end{array}$$ (1)

where *T* is air temperature, GDH_crit_ is heat requirement of the phenological event, and *T*_thr_ is threshold (base) temperature representing the lower thermal limit for development to occur. The value of *T*_thr_ is known to vary between tree species (Caffarra and Donnelly, 2011; Zhang *et al.*, 2022). This suggests that *T*_thr,_ should be estimated each time the model is fitted to data for a new species, cultivar, or bud type (Thompson and Clark, 2006; Man and Lu, 2010; Atagul *et al.*, 2022; Schiappacasse *et al.*, 2022; Bielenberg and Gasic, 2022)—henceforth, ‘ecophysiological approach’ and ‘ecophysiological GDH models’. Often, however, *T*_thr_ is fixed *a priori*, at the value of +5 °C, for instance (Cannell and Smith, 1983; Chen *et al.*, 2019; Wang *et al.*, 2020; Zohner *et al.*, 2020)—henceforth, ‘the climatological approach’ and ‘climatological GDH models’. In the climatological approach, only the heat requirement parameter, GDH_crit_, is estimated, so that only the steepness of the response, otherwise fixed, is allowed to vary (Supplementary Fig. S1B).

## Modelling analysis with theoretical phenological records

In process-based plant phenology models, a GDH model statistically fitted to observational phenological and temperature records is often taken as a proxy for the true physiological response (Cannell and Smith, 1983; Wang *et al.*, 2020). We examined the implications of that approach by using air temperature responses determined experimentally during quiescence (ecodormancy) (Box 1A; Supplementary Table S1) to develop theoretical phenological records and then fitting the GDH models to the phenological records generated (Box 1; Supplementary Fig. S2). Following Hunter and Lechowicz (1992), we generated the phenological records with models because in that way the correct air temperature response behind the ‘observations’ supposedly approximated by the GDH models is known with certainty. The models used in the data generation have a sound experimental basis (Zhang *et al.*, 2022, 2023), but that is immaterial for our study (Hunter and Lechowicz, 1992), as our study was intended to examine how well the GDH models can approximate the temperature regulation during quiescence in *any* plant species and bud type where the temperature response follows curves like the ones presented in Box 1A.

Box 1. **Outline of the study**

**Step 1. Experimentally based air temperature responses**

(A) The experimentally based air temperature responses of the rate of development during quiescence (Supplementary Table S1) were adopted from Zhang *et al*. (2022) for seedling leaf-out in four subtropical tree species growing in south-eastern China: *Castanopsis sclerophylla*, *Phoebe chekiangensis*, *Pseudolarix amabilis*, and *Torreya grandis*. For *Torreya*, an additional response for flowering in adult trees was available (Zhang *et al*., 2023). The response curves are sub-models in five corresponding process-based tree spring phenology models (Supplementary Fig. S2), where the other sub-models were also formulated on the basis of experimental research.

**Step 2. Generation of theoretical phenological records**

(B) By using weather station temperature records as input in computer simulations, the five process-based models were applied to generate theoretical records of the timing of the corresponding five phenological events in Hangzhou, southeastern China (30°08ʹN, 120°06ʹE) for 1958–2019. First, by using the method of Zohner *et al*. (2020), hourly temperatures were calculated for each day on the basis of records of daily minimum and maximum temperatures (http://data.cma.cn/). The hourly temperatures were then used as input for the five process-based models (Supplementary Fig. S2). The simulations were started on 23 November each year (Zhang *et al*., 2022, 2023).

**Step 3. Fitting the GDH model to the theoretical phenological records**

**Fig. 1.**
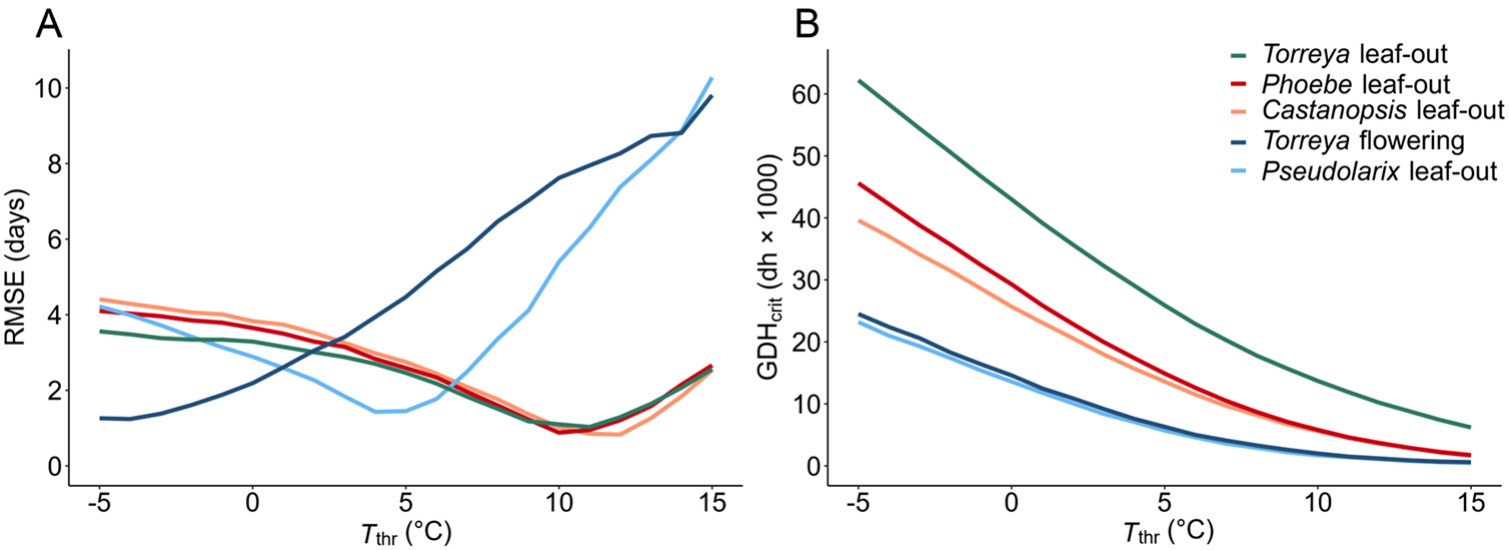
Performance of the growing degree hour (GDH) models with different values of the threshold (base) temperature (*T*_thr_) in analysing five theoretical springtime phenological records of subtropical trees between 1958 and 2019 in Hangzhou, southeastern China (Box 1B). Following the principle introduced by Hunter and Lechowicz (1992), we generated the theoretical phenological records by simulating the timing of the five springtime phenological events by means of five corresponding process-based models (Supplementary Fig. S2). In this way the true responses behind the generated data were known for certain. The performance of the GDH models was examined in simulations similar to the ones used for generating the theoretical observations, except that the experimentally based sigmoidal air temperature response (Box 1A) was replaced with one GDH model, with its particular value of *T*_thr_, at a time (Box 1C; Supplementary Fig. S2). (A) The root mean squared error (RMSE) of the prediction of the GDH model for the five phenological events as a function of the threshold temperature (*T*_thr_). (B) The heat requirement (GDH_crit_) of the five phenological events as a function of *T*_thr_. dh, degree hour.

The GDH model was fitted to each of the five theoretical phenological records by estimating the values of the model parameters threshold (base) temperature (*T*_thr_) and heat requirement (GDH_crit_) (Supplementary Table S2).

(C) In the first phase, a fixed value of *T*_thr_ was used. The phenological timing was simulated with the process-based model in ways otherwise similar to the ways of generating the theoretical phenological record, except that the original experimentally based temperature response [panel (A)] was now replaced with the GDH model with its particular fixed value of *T*_thr_ (Supplementary Fig. S2). This was repeated 21 times in the simulations by using 21 values of *T*_thr_, ranging with a step of 1 °C from –5 to +15 °C. Panel (C) shows the GDH model with *T*_thr_=+5 °C as an example. The first phase allowed us to determine the optimal value of GDH_crit_ for each *a priori* fixed *T*_thr_ minimizing the RMSE between the predicted and the ‘observed’ [generated, panel (B)] phenological timing (Fig. 1B) and also the dependence of the RMSE on *T*_thr_ (Fig. 1A).

(D) In the second phase, the optimal GDH model for the phenological event was determined by using the *T*_thr_ value that minimizes the RMSE (Fig. 1A). The resulting GDH models (blue lines) are compared with the corresponding experimentally based sigmoidal response (red curves) in the sub-panels (i)–(v) shown. Additionally, in sub-panel (vi), the GDH model for *Castanopsis* flowering and *Torreya* leaf-out are compared.

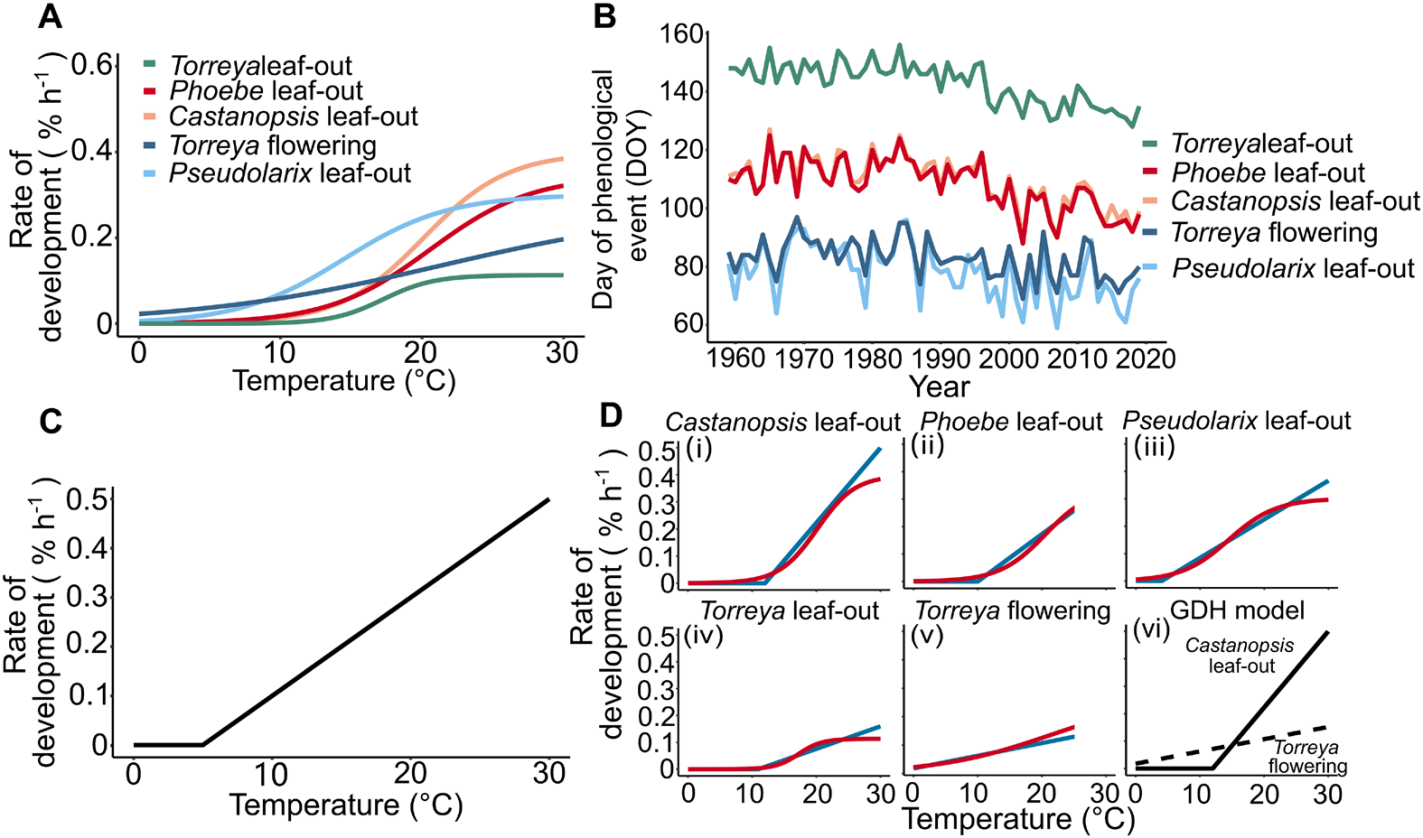

## Large variation in *T*_thr_ shows the need for ecophysiological growing degree hour models

For each *a priori* fixed value of *T*_thr_, we estimated the value of the heat requirement, GDH_crit_, by minimizing the root mean square error (RMSE) between the theoretical observations and the corresponding predictions of the GDH model (Fig. 1). The optimal *T*_thr_ minimizing the RMSE was −4 °C for *Torreya* flowering, +4 °C for *Pseudolarix* leaf-out, and in the range of +10 to +12 °C for the leaf-out in the other three species (Fig. 1A). Accordingly, this limited dataset, comprising only five phenological records, produced a range of 16 °C for the optimal *T*_thr_. This shows that the climatological GDH models using a fixed value of *T*_thr_ cannot realistically quantify the true physiological temperature response during quiescence for all species and bud types (Bielenberg and Gasic, 2022). However, despite the great variation in the true physiological value of *T*_thr_, when the common fixed value of +5 °C for *T*_thr_ was used in the climatological GDH model, then the RMSE stayed in the range of 1.2–4.1 d (Fig. 1A). Given the precision of phenological observations, such model accuracy is reasonable; so, our results suggest that the physiologically unrealistic climatological GDH models may be useful for predicting the timing of spring phenological events. Because of our limited data, however, that finding should be interpreted with caution, as earlier studies have emphasized the importance of using the true physiological response in making phenological predictions (Hänninen, 2006; Wolkovich *et al.*, 2021).

When the value of *T*_thr_ was estimated specifically for each of the five phenological records used, the resulting ecophysiological GDH models provided a sufficient proxy for the sigmoidal experimentally based response (Box 1D; Supplementary Table S2). This is also shown by the high accuracy of the ecophysiological GDH models with their optimal *T*_thr_ (RMSE between 0.7 and 1.2 d, Fig. 1A).

## Climatological growing degree hour models are needed for comparing heat requirements

The heat requirement, GDH_crit_, minimizing the RMSE decreased with the increasing *T*_thr_ used in the calculations (Fig. 1B). This inverse relationship has been reported before (e.g. Thompson and Clark, 2006; Atagul *et al.*, 2022), and it is actually a tautology, meaning that the correlation is inherent in the model: in natural conditions, the number of hours contributing to the degree hour accumulation automatically decreases with increasing *T*_thr_, and so does the increment of degree hours per any hour when they are also accumulated with a high value of *T*_thr_. Regardless of the value of *T*_thr_ used in the calculations, the ranking of the heat requirement among the five phenological records showed a consistent pattern (Fig. 1B) that followed the corresponding sequence of the five phenological records examined (Box 1B). This result, too, is a tautology because the later the accumulation of degree hours is stopped (i.e. the later the phenological event occurs), the higher is the number of degree hours accumulated. However, despite being based on two tautologies, the findings reported in Fig. 1B reveal a methodologically important conclusion: even though the climatological GDH models with an *a priori* fixed value for *T*_thr_ are physiologically unrealistic, they may be useful for climatological comparisons of the heat requirements of spring phenological events (Fig. 1B) (Chen *et al.*, 2019; Zohner *et al.*, 2020).

**Fig. 2.**
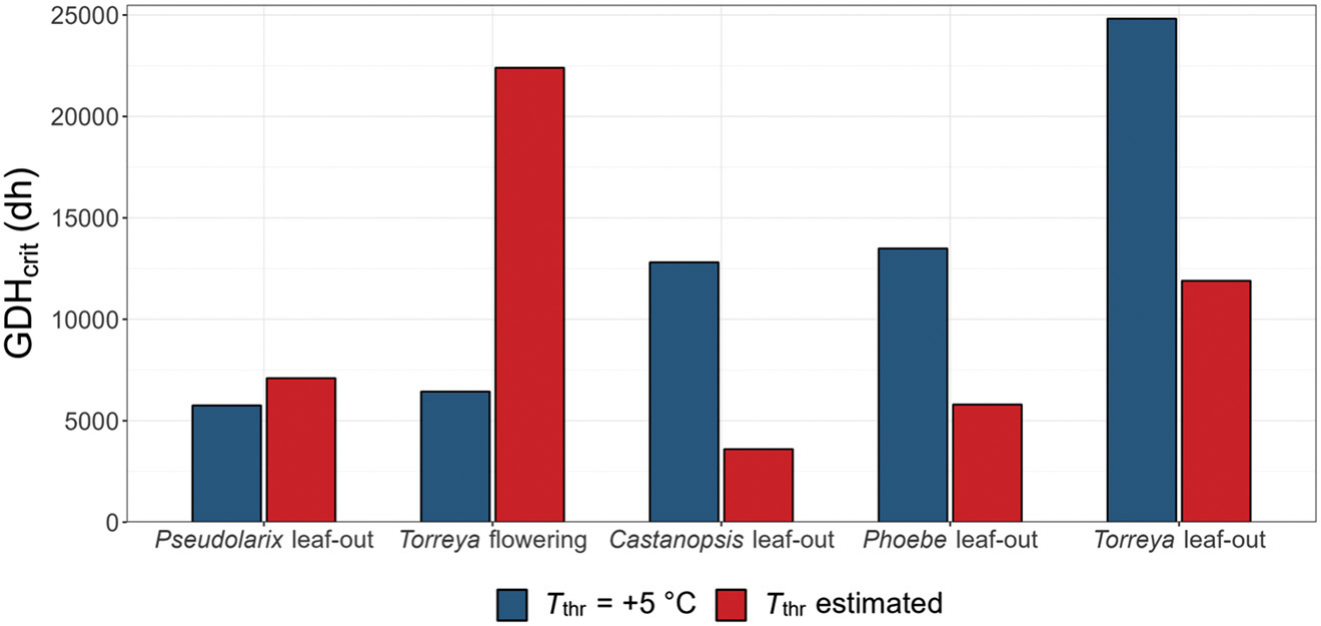
Comparison of heat requirements, GDH_crit_, estimated for five phenological events in subtropical trees by two methods when fitting the growing degree hour (GDH) model to theoretical phenological records: first, by fixing the threshold (base) temperature *a priori* at threshold temperature (*T*_thr_) of +5 °C (blue bars; see also Fig. 1B); and second, by estimating its value specifically for each of the five phenological records simultaneously when estimating the value of GDH_crit_ (red bars; Supplementary Table S2). The bars are presented from the left to the right, ordered according to increasing GDH_crit_ estimated by using the fixed value of *T*_thr_ (blue bars). dh, degree hour.

To be meaningful, the concept of heat requirement (GDH_crit_) presupposes that out of two phenological events, the one occurring earlier under the same conditions has the lower heat requirement. However, our earlier experimental results show that the relative earliness of *Torreya* flowering and *Castanopsis* leaf-out depends on the temperature: in experimental temperatures below +15 °C, *Torreya* flowering took place first, as indicated by its higher rate of development in those temperatures, but in temperatures above +15 °C, this was reversed (Box 1A). Accordingly, the respective two estimated GDH responses cross at +15 °C [Box 1D (vi)]. The corresponding GDH_crit_ estimated with the ecophysiological GDH model was much higher for *Torreya* flowering than for *Castanopsis* leaf-out (22 400 vs. 3600 degree hours, respectively; Fig. 2; Supplementary Table S2). However, despite its higher GDH_crit_, *Torreya* flowering always took place before *Castanopsis* leaf out in natural conditions (Box 1B) (Chamberlain and Wolkovich, 2021). This was because with its low value of *T*_thr_, the degree hours started to accumulate much earlier for *Torreya* flowering than for *Castanopsis* leaf-out with its high value of *T*_thr_. By this line of reasoning we see that the degree hours accumulated with different values of *T*_thr_ are not comparable, nor are the values of GHD_crit_ given in terms of the equations including the different *T*_thr_ values (Supplementary Table S2). Because of the lack of comparability, the ranking of the heat requirement, GDH_crit_, obtained with the ecophysiological approach was also different from that obtained with the climatological approach (Fig. 2). Most strikingly, when a fixed *T*_thr_ (e.g. +5 °C) was used in the climatological approach, *Torreya* flowering, together with *Pseudolarix* leaf-out, had the lowest heat requirement (Figs 1B, 2). As stated above, this reflects the relative earliness of these two phenological events (Box 1B). However, when the value of *T*_thr_ was estimated simultaneously with the estimation of GDH_crit_ in the ecophysiological approach, then *Torreya* flowering had the highest GDH_crit_ (Fig. 2).

The above reasoning shows that to get comparable results, the heat requirement for different phenological events should be determined with a physiologically unrealistic climatological GDH model applying a fixed value of *T*_thr_. This inference further shows that rather than a physiological parameter, the heat requirement is a climatological one (Fig. 1B), quantifying the overall earliness of the phenological event (Box 1B).

## Concluding remarks

Our results show that use of *a priori* fixed values for the threshold temperature, *T*_thr_, in temperature sum models runs a high risk of producing models of low biological realism. This emphasizes the need for experimental determination of the real ecophysiological temperature response and the use of species- and bud-type-specific values of *T*_thr_ in GDH models. However, ecophysiologically unrealistic climatological GDH models with a fixed *T*_thr_ are needed when one wishes to compare the heat requirements of different species or bud types. This paradoxical result shows that the concept of heat requirement, GDH_crit_, is a climatological rather than a physiological one.

## Supplementary data

The following supplementary data are available at *JXB* online.

Fig. S1. Two representations of the growing degree hour (GDH) model.

Fig. S2. Overall rationale of the study.

Table S1. Equation and parameter values of the five sigmoidal temperature responses used in the present study.

Table S2. Parameter values of the five growing degree hour (GDH) models fitted to the five corresponding theoretical phenological records in the present study.

erae363_suppl_Supplementary_Figures_S1-S2_Tables_S1-S2

## Data Availability

Data are available from the corresponding author upon request.
