# Supplementary material for: Temperature sum models in plant spring phenology studies: two commonly used methods have different fields of application
Source: J Exp Bot. 2024 Aug 27;75(19):6011–6. doi: 10.1093/jxb/erae363 (PMC11480661; doi:10.1093/jxb/erae363)
Supplement: erae363_suppl_Supplementary_Figures_S1-S2_Tables_S1-S2 [file erae363_suppl_supplementary_figures_s1-s2_tables_s1-s2.pdf]

Supplementary data

Temperature sum models in studies of plant spring phenology: two commonly used methods have different application fields

Rui Zhang, Fucheng Wang, Jinbin Zheng, Lei Chen, Heikki Hänninen, and Jiasheng Wu

Supplementary data figures

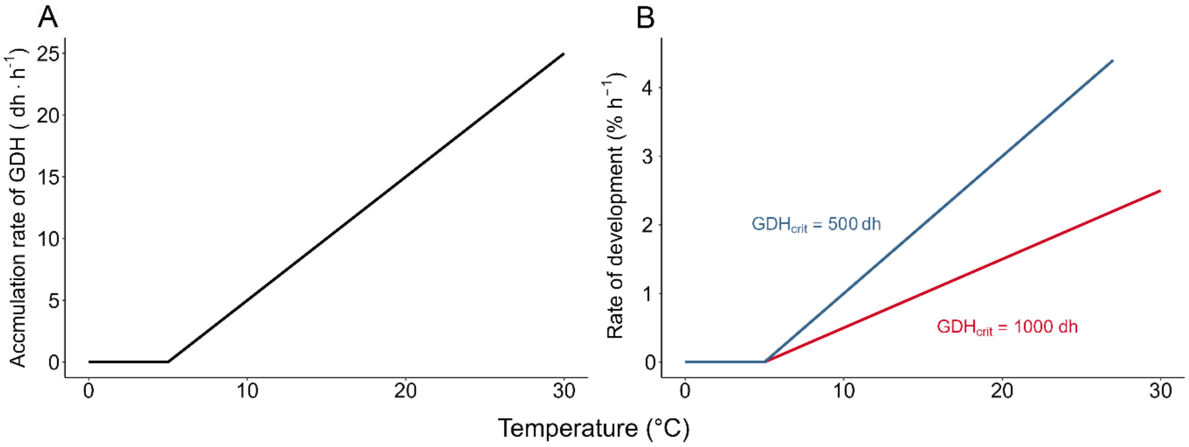

Supplementary Fig. S1. Two representations of the Growing Degree Hour (GDH) model with the threshold (base) temperature of T_{thr} = +5 °C. (A) The original representation. The vertical axis indicates the accumulation rate of GDH, but not the rate of development during quiescence. (B) A modified representation, where the accumulation rate of GDH is multiplied by 100/GDH_{crit}, where GDH_{crit} = the heat requirement of the phenological event (Hänninen, 2016). The vertical axis indicates the rate of development during quiescence as the percentage during one hour of developmental events out of the total development required for the springtime phenological event to occur.

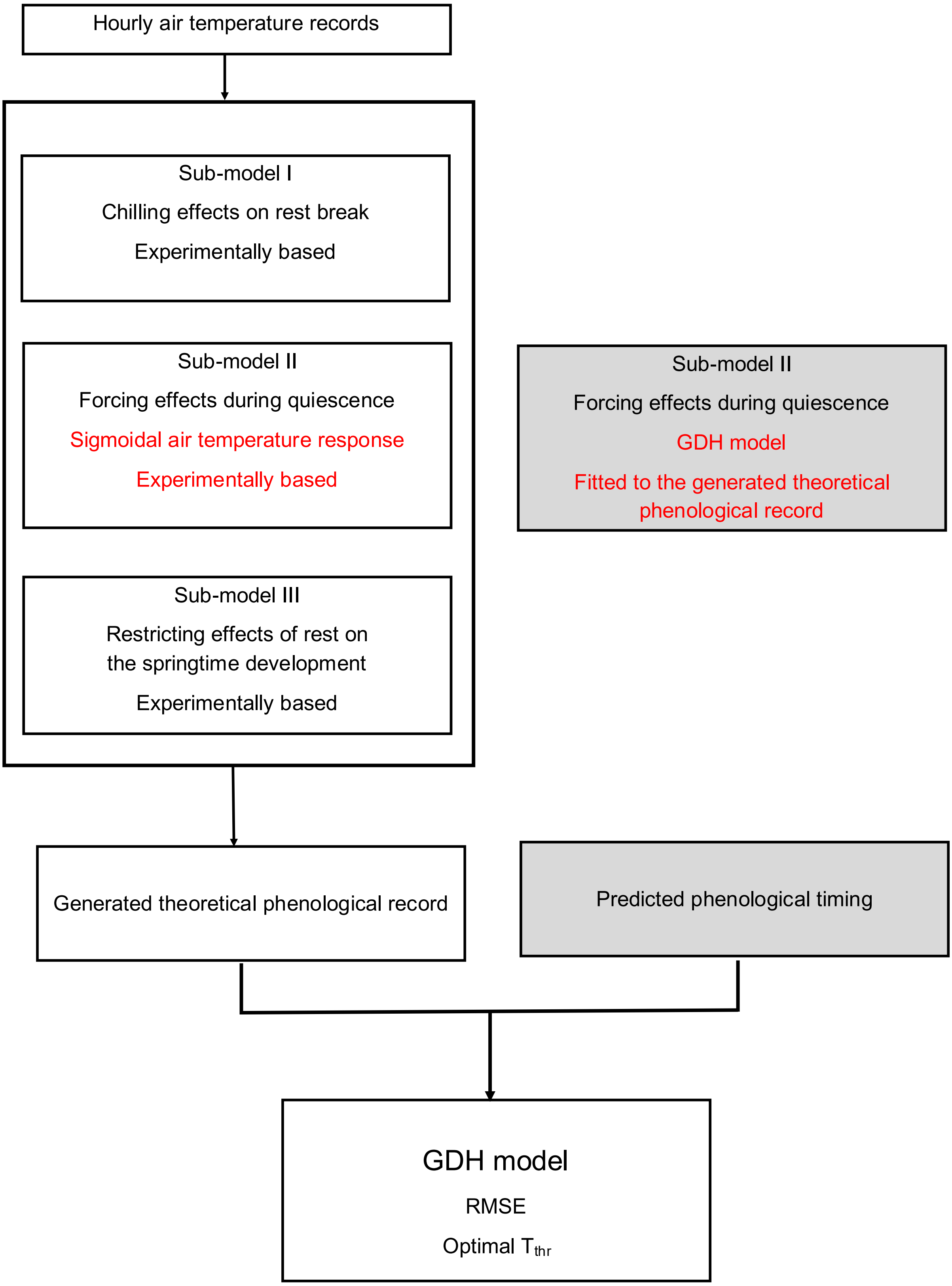

Supplementary Fig. S2. Overall rationale of the study. Left-hand side: process-based tree spring phenology models were first used for generating theoretical phenological records (Box 1B in the main paper). Subsequently, the simulations were carried out otherwise similarly, but

the experimentally based sigmoidal air temperature response (Box 1A in the main paper) was replaced by one of the 21 Growing Degree Hour (GDH) models at a time (Box 1 C in the main paper); changes for these simulations are indicated on the right-hand side. The GDH models were tested by comparing their predictions with the generated phenological records.

Supplementary data tables

Supplementary Table S1. Equation and parameter values of the five sigmoidal temperature responses (Box 1A in the main paper) used in the present study in the five corresponding process-based tree phenology models when generating the theoretical phenological records (Box 1B in the main paper). The equation is given at the bottom of the table, T = hourly temperature. The models were adopted from Zhang *et al.* (2022, 2023).

	**Parameter**		
**Phenological event**	**a (% h^{-1})**	**b (°C)**	**c (°C)**
Torreya flowering	0.2709	-8.8756	21.3366
Torreya leaf-out	0.1130	-1.8091	17.2439
Pseudolarix leaf-out	0.3008	-3.6984	14.4639
Castanopsis leaf-out	0.3984	-2.9868	20.0907
Phoebe leaf-out	0.3451	-3.6026	20.5672
	$Rate\ of\ development(T) = \frac{a}{1 + e^{\frac{1}{b}(T-c)}}$		

Supplementary Table S2. Parameter values of the five physiological Growing Degree Hour (GDH) models (Box 1D; and Eqn. (1) in the main paper) fitted in the present study to the five corresponding theoretical phenological records (Box 1B in the main paper). T_{thr} = threshold (base) temperature, GDH_{crit} = heat requirement of the phenological event, dh = degree hour unit.

**Phenological event**	**T_{thr} (°C)**	**GDH_{crit} (dh)**
Torreya flowering	-4	22 400
Torreya leaf-out	+11	11 900
Pseudolarix leaf-out	+4	7 100
Castanopsis leaf-out	+12	3 600
Phoebe leaf-out	+10	5 800
